# The Kaiser Permanente Research Bank Cancer Cohort: a collaborative resource to improve cancer care and survivorship

**DOI:** 10.1186/s12885-022-09252-6

**Published:** 2022-02-25

**Authors:** Heather Spencer Feigelson, Christina L. Clarke, Stephen K. Van Den Eeden, Sheila Weinmann, Andrea N. Burnett-Hartman, Sarah Rowell, Shauna Goldberg Scott, Larissa L. White, Monica Ter-Minassian, Stacey A. A. Honda, Deborah R. Young, Aruna Kamineni, Terrence Chinn, Alexander Lituev, Alan Bauck, Elizabeth A. McGlynn

**Affiliations:** 1 Institute for Health Research, Kaiser Permanente, 2550 S. Parker Rd, Suite 200, Aurora, CO 80014 USA; 2 Division of Research, Kaiser Permanente, 2000 Broadway, Oakland, CA 94612 USA; 3 Center for Health Research, Kaiser Permanente, 3800 N. Interstate Ave, Portland, OR 97227 USA; 4 Kaiser Permanente Program Office, 1800 Harrison, 16th floor, Oakland, CA 94612 USA; 5 Mid-Atlantic Permanente Research Institute, Kaiser Permanente, 2101 East Jefferson St, 3 West, Rockville, MD 20852 USA; 6 Center for Integrated Healthcare Research and Hawai’i Permanente Medical Group, Kaiser Permanente, 501 Alakawa St Suite 201, Honolulu, HI 96817 USA; 7 Department of Research and Evaluation, Kaiser Permanente, 100 S. Los Robles Avenue, Pasadena, CA 91101 USA; 8 Kaiser Permanente Washington Health Research Institute, 1730 Minor Ave Suite 1600, Seattle, WA 98101 USA; 9 Kaiser Permanente Research Bank, Kaiser Permanente, 1795 A Second St, Berkeley, CA 94710 USA; 10 Kaiser Permanente Research & Quality Measurement and Kaiser Permanente Research Bank, 100 S. Los Robles, 3rd floor, Pasadena, CA 91101 USA

**Keywords:** Cancer, Survivorship, Cohort study, Disparities, Genetic

## Abstract

**Background:**

The Kaiser Permanente Research Bank (KPRB) is collecting biospecimens and surveys linked to electronic health records (EHR) from approximately 400,000 adult KP members. Within the KPRB, we developed a Cancer Cohort to address issues related to cancer survival, and to understand how genetic, lifestyle and environmental factors impact cancer treatment, treatment sequelae, and prognosis. We describe the Cancer Cohort design and implementation, describe cohort characteristics after 5 years of enrollment, and discuss future directions.

**Methods:**

Cancer cases are identified using rapid case ascertainment algorithms, linkage to regional or central tumor registries, and direct outreach to KP members with a history of cancer. Enrollment is primarily through email invitation. Participants complete a consent form, survey, and donate a blood or saliva sample. All cancer types are included.

**Results:**

As of December 31, 2020, the cohort included 65,225 cases (56% female, 44% male) verified in tumor registries. The largest group was diagnosed between 60 and 69 years of age (31%) and are non-Hispanic White (83%); however, 10,076 (16%) were diagnosed at ages 18–49 years, 4208 (7%) are Hispanic, 3393 (5%) are Asian, and 2389 (4%) are Black. The median survival time is 14 years. Biospecimens are available on 98% of the cohort.

**Conclusions:**

The KPRB Cancer Cohort is designed to improve our understanding of treatment efficacy and factors that contribute to long-term cancer survival. The cohort’s diversity - with respect to age, race/ethnicity and geographic location - will facilitate research on factors that contribute to cancer survival disparities.

## Background

Between 2016 and 2040, the projected prevalence of cancer survivors in the United States (U.S.) will increase from an estimated 15.5 million (approximately 4.8% of the population) to 26.1 million survivors (nearly 7% of the total population) across all age groups [1]. With advances in early detection and the increasing effectiveness of cancer treatments, long-term survivorship is becoming a reality for more than half of those diagnosed with cancer. Currently, an estimated 64% of all survivors have lived at least 5 years beyond diagnosis and 40% have lived at least 10 years beyond diagnosis [2, 3].

In 2015, then President Obama announced the Precision Medicine Initiative [4] with the intent to accelerate research that integrates molecular and genomic information into medical care. This was followed in 2016 by the Enactment of the Cancer Moonshot within the twenty-first Century Cures Act, to accelerate progress to prevent, diagnose and treat cancer [5]. While these initiatives have provided enthusiasm and funding for the identification of highly targeted agents that improve cancer treatment efficacy, to date, the focus on precision medicine has largely ignored other factors that may influence treatment and survival, such as co-morbid conditions, medication use, and lifestyle habits. The promise of precision medicine should include medical care that goes beyond the acute treatment period and maximizes long-term quality of life for cancer survivors.

Kaiser Permanente (KP) is an ideal environment to study the spectrum of cancer care and long-term survival because it provides comprehensive medical care, including cancer care, to a large and diverse membership. For decades, KP has used electronic health records (EHR) to track patient care, including preventive services, diagnoses, and treatments. The vast majority of KP members diagnosed with cancer receive all their follow-up care within KP facilities and the details of that care are captured in the EHR. The KP Research Bank (KPRB), a research resource within KP, is seeking to collect biospecimens and surveys from at least 400,000 adult members across the U.S. and linking that information to the EHR. Within the KPRB, we have developed a Cancer Cohort to specifically address issues related to cancer survival, and to understand how genetic, lifestyle, and environmental factors impact cancer treatment, treatment sequalae, and prognosis. In this paper, we describe the design and implementation of the KPRB Cancer Cohort, present characteristics of the cohort after 5 years of enrollment and discuss future directions.

## Methods

### The Kaiser Permanente Research Bank

KP provides health care for approximately 12.5 million individuals across eight regions: Colorado, Georgia, Hawai’i, Mid-Atlantic, Northern California, Northwest, Southern California, and Washington. All KP members aged 18 and older whose preferred language is English or Spanish are eligible to join the KPRB. Launched in May 2016, the KPRB is built on an existing repository (Research Program on Genes, Environment and Health, or RPGEH) of approximately 220,000 KP members with data and biospecimens collected primarily between 2007 and 2011 from members of KP Northern California, and to a smaller extent, Southern California [6]. The KPRB consists of three cohorts: a general cohort, a pregnancy cohort [7], and a cancer cohort. The primary recruitment mode is by email invitation; up to 5 invitations to enroll are sent. KP Washington members are invited to participate by postal mail letters only, and a small percentage of members across the remaining regions who are not registered on the member portal also receive mailed invitations. Several regions also use in-person recruitment to increase enrollment among racially and ethnically diverse members. KP members can also initiate enrollment without a specific initiation by visiting the KPRB website (https://researchbank.kaiserpermanente.org/). Approximately 396,000 KP members have consented to join the KPRB as of July 2021, and biospecimens have been collected on approximately 371,000 adults (94% of those consented).

Participation in the KPRB involves completing the consent form, survey, and providing a saliva or blood sample. Except for those who request paper materials, members enroll by visiting the KPRB website and completing an online consent and survey. This triggers the placement of a blood draw order for specimen collection. The consent form gives permission to access the EHR, ascertain and store clinically collected specimens (including tissue biopsies and surgical resections), allow future contact, as well as use of survey data, DNA, and other blood components for research. The baseline survey collects information on demographics; neighborhood walkability/safety; work exposures and shift work; general health (PROMIS 10); social media use; chronic pain; diet, physical activity, sleep, and sedentary behavior; multi-vitamin, vitamin D, calcium, and non-steroidal anti-inflammatory drugs; personal and family history, including cancer; alcohol, tobacco, marijuana, and opiate drugs; stress, isolation, support, and discrimination; reproductive history in women; urinary function and erectile dysfunction in men; genetic testing, sigmoidoscopy and colonoscopy; and health literacy. The KPRB recruitment protocol was approved by the Institutional Review Board (IRB) in KP Mid-Atlantic States which serves as the single IRB for KPRB recruitment.

### Biospecimen collection

Blood samples are collected at the member’s convenience at any KP clinical laboratory and include up to 4 ml of whole blood (collected in one EDTA tube) for DNA and up to 8.5 ml of serum (in two SST tubes). Specimens are collected and shipped Monday through Friday (and Saturday in some regions) by overnight courier to the biorepository in Berkeley, CA. Specimens are tracked, and “needle to freezer” time is recorded for each sample; 56 and 83% are processed and frozen within 48 and 72 h of collection, respectively. DNA is extracted from whole blood using an automated Thermo Kingfisher system. DNA samples are dried down in Micronic tubes with Biomatrica DNAstable Plus storage medium for ambient storage using Thermo Savant Explorer SpeedVac system. DNA samples are re-hydrated, quantified, and normalized prior to distribution for research projects. Serum and whole blood are transferred into Micronic tubes for freezing and long-term storage at -80^o^ C.

### Linkage to electronic health data

Each KP region captures EHR data for research using a standardized, structured common data model, known as the Virtual Data Warehouse (VDW). The VDW includes comprehensive data on patient characteristics, diagnoses, medical procedures, and medication use [8, 9]. EHR data from KPRB members are updated in the KPRB data repository every 6 months. Most data are current at the time of the update, but data that come from outside sources, such as vital status and cause of death, may lag up to 12 months. Sources of vital status data include: the EHR, state vital statistics, and local (e.g. tumor registry) or national (e.g. Social Security Administration, National Death Index) registries.

### The Cancer Cohort

The KPRB Cancer Cohort uses the same website, consent form, and blood draw protocol as the general cohort. The Cancer Cohort survey includes all questions from the general cohort with additional questions specifically relating to cancer about genetic testing, cancer screening, and family history of specific cancers. Study materials are available in English and Spanish (beginning in 2018) languages.

We developed recruitment strategies and materials specifically designed to increase the enrollment of people with cancer. First, we developed a rapid case ascertainment (RCA) algorithm (described below) to identify cancer cases shortly after diagnosis. Second, from January–June of 2021, we used tumor registry and EHR data to identify members with a history of cancer (regardless of when the diagnosis occurred) and invited them to enroll using recruitment materials with content specifically about cancer survivorship. Some regions used direct outreach to oncology departments and care navigators, and in-clinic recruiters to increase enrollment; patients received flyers about enrolling into the KPRB in materials provided during their oncology visits. In addition to these methods, cancer cases are identified from members of the general cohort who report a history of cancer, and incident cases that arise in the general cohort after enrollment. Cancer stage is defined using Surveillance, Epidemiology and End Results (SEER) general summary stage [10] as follows: in-situ, localized, regional, distant, benign/borderline, unknown, or missing. Study enrollment was paused in March 2020 due to the SARS-CoV-2 pandemic. Cancer cases identified using the RCA were invited to enroll again starting in October 2020.

### Development of the rapid case ascertainment (RCA) algorithm

To maximize the value of this resource, we aimed to identify and enroll cancer cases shortly after diagnosis, allowing us to minimize survival bias and, in some cases, collect specimens prior to the start of treatment.

**Fig. 1:**
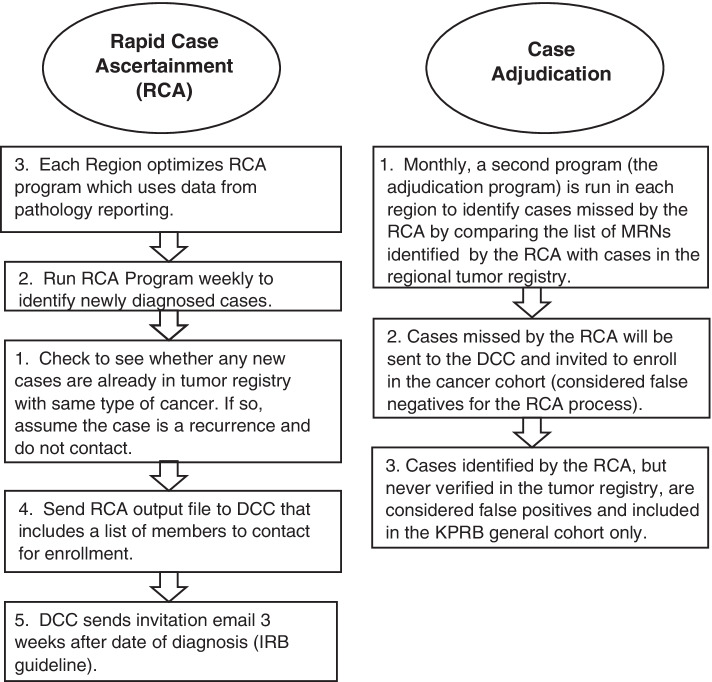
Diagram of the Rapid Case Ascertainment Process. Rapid Case Ascertainment (RCA) process uses pathology data to identity new cancer cases within days of diagnosis. Probable cancer cases are adjudicated using linkage to the tumor registry data in each Kaiser Permanente region. Abbreviations: DCC: Data Coordinating Center; IRB: Institutional Review Board; MRN: Medical Record Number

KP maintains regional tumor registries in 6 of 8 KP regions to identify and track cancers that were diagnosed and treated within the health system (2 regions have relied on linkage to SEER registries) [10]. While tumor registries are considered the gold standard for the identification of incident cancers, data in the tumor registries can lag 6–18 months behind the date of diagnosis because tumor registrars identify potentially eligible cases and then manually abstract to confirm the diagnosis and ascertain the state or federally required data elements about the cancer [11]. Thus, we developed RCA algorithms to identify new cancer cases within days of diagnosis and assign an anatomical site with a high degree of accuracy using data pulled directly from electronic pathology files (Fig. 1).

Because each KP region uses different software and systems for pathology data, region-specific approaches to RCA were required. However, each region with a tumor registry followed a similar process in algorithm development. The tumor registry was used as the gold standard, and the best data source from the pathology department was determined. In some regions, a file is sent directly from pathology to the tumor registrars, and when available this was the file of choice. Tumor registry data from a prior year was used to develop the algorithm to ensure that the data were complete. Once the pathology data were acquired, the development of the algorithm was iterative; computer code (using SAS, version 9.4 (SAS Institute, Cary NC)) was developed to identify cancer cases from non-cancer pathology specimens. Sensitivity and specificity were calculated for each iteration, and chart review was used as necessary to understand at each iteration why false positive and false negative cases were mis-specified. Because the algorithms were intended to identify people with a high probability of cancer, we focused on achieving high specificity, but also evaluated the loss of sensitivity for each incremental gain of specificity to make the algorithm as robust as possible. As a final step, the algorithm was tested on “live” data prior to launch, and chart review was conducted on a small number of records (< 50 in each region) to verify that the algorithm was performing as expected.

Two regions (Northwest and Northern California) used the Systematized Nomenclature of Medicine (SNOMED) coding system [12]. From SNOMED, we used a selection of morphology, “M” codes that indicated cancer and the corresponding topography/anatomical terms “T” from the same pathology report. The “T” codes were used to identify the anatomical site from the pathology report and created a “probable cancer site” variable within the algorithm. The KP Colorado tumor registry uses the E-Path software system (https://www.inspirata.com/solutions/cancer-registry-automation/). Working with the local tumor registry at KP Colorado, we developed a direct feed from E-Path for the RCA system. A similar process was adopted at KP Mid-Atlantic States, which uses a direct feed from the EHR and CoPath Plus to the Cancer Alert system (CAS) which scans both feeds for defined International Classification of Diseases (ICD) codes and keywords*.* The E-Path and CAS algorithms are the primary source of records by tumor registrars. KP Southern California used the software system, Linguamatics product I2E (https://www.linguamatics.com/products/i2e), to identify cases from pathology reports. Linguamatics (Marlborough, MA) is a commercial software coding product to mine text using natural language processing (NLP). Linguamatics developed the NLP coding, and it was distributed to KP Southern California to test and refine on their pathology reports. KP Hawai’i developed a program to identify cancer cases from the EHR Problem List and claims data. KP Washington also used the Problem List to identify recent cancer diagnoses among enrolled members attributed to the internal delivery system and residing within the 13-county Seattle-Puget Sound SEER registry catchment area. Among these members with Problem List-noted cancer diagnoses, pathology records with cancer-related text strings (using a list provided by the Seattle-Puget Sound SEER registry) and specimen collection dates within 60 days prior to the cancer diagnosis dates noted on the Problem List were identified. Pathology records with non-melanoma skin cancers, cancers from primary site metastasis, or those with clear cancer-negating text were excluded.

The development of the RCA process in each KP region was phased in over several months. The first regions (Northern California, Colorado, Northwest) launched in May 2016, Hawai’i launched in June 2016, Southern California launched in November 2016, Mid-Atlantic States launched in 2017, and Washington launched in 2019. The RCA was launched in each region after the algorithm was optimized and tested. After launch, the process is largely automated. Every week the RCA program runs in each region to identify new cancer cases and generates a data file that is sent to the KPRB Data Coordinating Center (DCC). The DCC checks the file to determine the member’s eligibility for recruitment outreach (enrolled in KP, alive, at least 18 years of age, and not on any “do not contact” list) and then invites the member to participate in the study 3 weeks after the date of diagnosis. The IRB required a three-week window after diagnosis prior to study contact. The member is contacted via email or postal mail with an invitation that indicates they are being contacted to join the KPRB because they ‘recently received a procedure or test’. The recruitment materials do not indicate that the member has, or may have, cancer. This was done to protect patient privacy and to avoid alarming patients who turn out to be false positives.

### Case adjudication

Case adjudication relies on the regional or SEER tumor registries to accomplish three critical functions: (1) validate the results of the RCA process, (2) identify incident cases missed by the RCA, and (3) identify prevalent and incident cases in the KPRB general and pregnancy cohorts. Beginning at least 6 months after the Cancer Cohort is launched in a region, members who have enrolled in the Cancer Cohort are compared against the tumor registry. In most regions, case adjudication is done monthly. Data from the tumor registry are used to confirm the type of cancer, and whether the case identified by the RCA is incident or a cancer recurrence. Members missed by the RCA algorithm, and who are not already enrolled in the KPRB, are sent an invitation to enroll. Cancer cases identified from the general cohort during adjudication are flagged for inclusion in the Cancer Cohort. Because these members already have donated biospecimens and completed a survey, they are not contacted further. Finally, members identified by the RCA algorithm who are not in the tumor registry within 12 months are considered false positives; these members are no longer considered part of the Cancer Cohort and become part of the general cohort.

## Results

The positive predictive value (PPV) of the RCA algorithm at each site ranges between 80 and 99%, indicating that we have successfully minimized inviting people who have not been recently diagnosed with cancer (i.e., the false positive rate is low). Response rates for both the RCA process over the entire recruitment period and the cancer survivor outreach which occurred from January–June 2021 to KP members with a history of cancer are shown in Table 1. The response rate from RCA was 9.8% overall and for survivors was 8.8%; however, we observed important differences by race/ethnicity. Response rates in non-Hispanic Whites were 14.6 and 15.3%, respectively, but response rates from RCA were less than 5% for Blacks, Hispanics, Native Hawaiian/Pacific Islanders, and those with missing race/ethnicity information. Among survivors, response rates were also less than 5% for all the above groups as well as American Indian/Alaska Natives and Asians.

**Table 1:** Kaiser Permanente Research Bank Cancer Cohort response rates by method of case identification

	Cases identified by Rapid Case Ascertainment			KP members with a history of cancer in the EHR		
	Number Contacted	Number Consented	% Consented	Number Contacted	Number Consented	% Consented
Sex^a^						
Female	71,550	7439	10.4%	126,372	11,250	8.9%
Male	56,411	5115	9.1%	92,616	7956	8.6%
Age at Diagnosis (years)^b^						
< 50	28,466	1956	6.9%			
50–59	32,372	2460	7.6%			
60–69	48,694	4304	8.8%			
70–79	36,287	3462	9.5%			
≥ 80	17,844	1118	6.3%			
Race/Ethnicity^c^						
White, non-Hispanic	64,415	9394	14.6%	95,018	14,551	15.3%
Hispanic	24,558	1163	4.7%	47,722	1767	3.7%
Asian	16,607	952	5.7%	28,263	1116	3.9%
Black	14,799	564	3.8%	31,603	1061	3.4%
Multi-Racial	2431	226	9.3%	6293	329	5.2%
Native Hawaiian/Pacific Islander	2073	84	4.1%	3573	105	2.9%
American Indian/Alaska Native	687	55	8.0%	1522	69	4.5%
Other/Unknown	2394	117	4.9%	5005	210	4.2%

^a^Excludes 14 people with missing information on sex; ^b^Age at diagnosis was not known for all members with a history of cancer at the time of invitation to participate; ^c^Race/ethnicity information was obtained from the EHR and may not reflect self-reported data

Table 2 shows the characteristics of the 65,225 KPRB cancer cases that have been verified in the tumor registries through 2020 (exact dates vary by KP region). This does not include cases who were recently identified by the RCA process that have yet to be verified since tumor registry data can lag up to 18 months behind identification by the RCA. Characteristics are shown for all cases, and for those diagnosed within 6 months (prior to) enrollment, incident cases (after KPRB enrollment), and prevalent cases diagnosed more than 6 months prior to enrollment. Most members diagnosed within 6 months prior to enrollment were identified through the RCA process. Because the KPRB was built upon the preexisting RPGEH cohort of nearly 200,000 people, most members were diagnosed more than 6 months prior to enrollment (*N* = 37,309). However, there are 21,157 incident cases, and an additional 6759 diagnosed within 6 months of KPRB enrollment. Fifty-six percent of the cases are female (*N* = 36,492) and 44% are male (*N* = 28,733). While most members were diagnosed over the age of 60 years, 10,076 (16%) were diagnosed between 18 and 49 years of age. Most participants are non-Hispanic White (83%), but the cohort also includes 4208 (7%) Hispanic, 3393 (5%) Asian, and 2389 (4%) Black participants. The median survival time is 14 years (currently 674,136 total person-years of follow-up) and 17,554 participants have died (data not shown). Blood or saliva specimens are available on 98% of the Cancer Cohort.

**Table 2:** Demographic Characteristics of Cancer Cohort members verified in the tumor registry through 2020

	Incident within 6 months prior to KPRB enrollment (*N* = 6759)		Incident after KPRB enrollment (*N* = 21,157)		Prevalent (Diagnosed more than 6 months prior to KPRB enrollment) (*N* = 37,309)		Total (*N* = 65,225)	
	N	%	N	%	N	%	N	%
Sex								
Female	4075	60%	11,152	53%	21,265	57%	36,492	56%
Male	2684	40%	10,005	47%	16,044	43%	28,733	44%
Age at Diagnosis (years)								
< 18					94	< 1%	94	< 1%
18–49	1124	17%	950	5%	8002	22%	10,076	16%
50–59	1498	22%	1979	9%	9366	25%	12,843	20%
60–69	2314	34%	5791	27%	12,177	33%	20,282	31%
70–79	1419	21%	7405	35%	6398	17%	15,222	23%
≥ 80	404	6%	5032	24%	1272	3%	6708	10%
Year of Diagnosis								
Before 2015	966	14%	11,017	52%	33,300	89%	45,283	70%
2015	38	< 1%	1740	8%	1664	5%	3442	5%
2016	1398	21%	1842	9%	1043	3%	4283	7%
2017	1725	26%	2359	11%	727	2%	4811	7%
2018	1804	27%	2521	12%	497	1%	4822	7%
2019	796	12%	1563	7%	73	< 1%	2432	4%
2020	32	< 1%	115	1%	5	< 1%	152	< 1%
Race/Ethnicity^a^								
White, non-Hispanic	5213	77%	18,381	87%	30,712	83%	54,306	83%
Hispanic	595	9%	1068	5%	2545	7%	4208	7%
Asian	485	7%	951	5%	1957	5%	3393	5%
Black	289	4%	587	3%	1513	4%	2389	4%
Native Hawaiian/Pacific Islander	18	< 1%	38	< 1%	82	< 1%	138	< 1%
American Indian/Alaska Native	21	< 1%	38	< 1%	74	< 1%	133	< 1%
Multi-Racial/Other	106	2%	77	< 1%	359	1%	542	1%
Missing	32	1%	17	< 1%	67	< 1%	116	< 1%

^a^Self-reported race/ethnicity (may differ from information in the electronic health record)

**Fig. 2:**
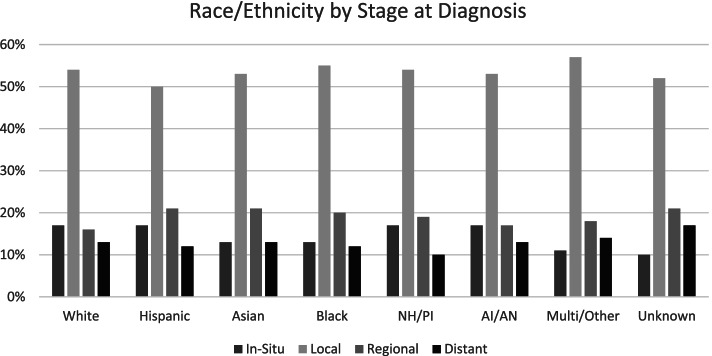
Stage at Diagnosis by Race/Ethnicity of Cancer Cohort Members. NH/PI: Native Hawaiian/Pacific Islander; AI/AN: American Indian/Alaska Native; Multi/Other: Multi-Racial/or other race/ethnicity

Table 3 shows the most common types of cancer included in the KPRB by sex, and the distribution of cases by stage at diagnosis. The most common types of cancer are breast, prostate, melanoma, and colorectal. Most cancers are diagnosed at the localized stage (46% of women and 54% of men), whereas 11% of cancers in women and 14% in men are diagnosed at the distant stage. This distribution reflects, in part, the high screening rates of KP members for breast, colorectal, and cervical cancers. We observed little variation in stage at diagnosis by race/ethnicity across cancer types (Fig. 2). Between 50 and 57% of cases were diagnosed with localized disease, and 10–17% of cases were diagnosed with distant metastases.

**Table 3:** Anatomic Site and Summary Stage at Diagnosis for Cancer Cohort Members by Sex

	Total (*N* = 65,225)	Females (*N* = 36,492)		Males (*N* = 28,733)	
Anatomic Site	N	N	%	N	%
Breast	15,080	14,962	99%	118	1%
Prostate	10,509			10,509	100%
Melanoma	9048	4280	47%	4768	53%
Colorectal	4717	2386	51%	2331	49%
Head and Neck	3055	1615	53%	1440	47%
Leukemia	3042	1343	44%	1699	56%
Lung	2917	1661	57%	1256	43%
Bladder	2416	538	22%	1878	78%
Cervical	2212	2212	100%		
Endometrial	1994	1994	100%		
Lymphoma	1751	869	50%	882	50%
Kidney and Ureter	1562	569	36%	993	64%
Brain and Other Nervous System	1550	1006	65%	544	35%
Pancreatic and Biliary	1371	651	47%	720	53%
Upper Gastrointestinal Tract	991	345	35%	646	65%
Other^a^	3010	2061	68%	949	32%
	Total (*N* = 65,225)	Females (*N* = 36,492)		Males (*N* = 28,733)	
Summary Stage^b^	N	N	%	N	%
In-Situ	10,261	6897	19%	3364	12%
Local	32,354	16,864	46%	15,490	54%
Regional	10,243	6256	17%	3987	14%
Distant	7951	3893	11%	4058	14%
Benign/Borderline	1263	855	2%	408	1%
Unknown	1983	981	3%	1002	4%
Missing	1170	746	2%	424	1%

^a^Other includes cancers of the following systems and sites: eye, musculoskeletal, genitourinary otherwise not listed, gynecologic otherwise not listed, condition-specific sarcomas, and unspecified/unknown cancers. ^b^SEER general summary stage [8]

## Discussion

The KPRB Cancer Cohort is a unique resource to study the spectrum of cancer care and survival. A key advantage of this cohort is the linkage of EHR records to biospecimens. Historically, member retention in KP has been high, facilitating passive follow-up for research studies using the EHR. A report from the Cancer Research Network that included data from 4 KP regions found that 70% of cancer cases had been health plan members for at least 5 years prior to their diagnosis and only 20% had disenrolled within 5 years after diagnosis [13]. The longitudinal data, both prior to and after cancer diagnosis provides a wealth of information on screening, premalignant lesions, specific treatment modalities, information on medication use and comorbidities, and cancer risk factors such as BMI and smoking status. The EHR can be combined with the KPRB survey data to provide a comprehensive source of risk factor and lifestyle information. Tissues samples are not formally collected as part of the KPRB but clinically collected surgical tissue samples are stored at each KP region and their use is covered in the KPRB consent form. Finally, the Cancer Cohort can be compared against the KPRB general cohort, that currently includes over 330,000 adults with no history of cancer. Detailed information about available data and the application process to use the KPRB resource (for both KP-affiliated and non-KP affiliated investigators) are available on the website (https://researchbank.kaiserpermanente.org/our-research/for-researchers).

An important strength of the Cancer Cohort is its diversity with respect to age, race/ethnicity, and geographic location. The cohort currently includes over 10,000 people diagnosed under age 50 and significant numbers of Hispanic (*N* = 4208), Asian (*N* = 3393), and Black (*N* = 2389) members. Geographic disparities in life expectancy among U.S. counties are large and increasing [14]. Socioeconomic, behavioral and health care factors all contribute to the variation in life expectancy across the U.S. Our cohort includes KP members from across the U.S. (including Hawai’i), providing an opportunity to investigate differences in cancer care and survival by geographic location among an insured population with access to health care.

Including all cancers, rather than targeting enrollment to a specific set of cancers, offers several advantages, including making in-clinic recruitment and study promotion inclusive of all KP members. Providers and clinic staff can discuss the study without considering whether a particular type of cancer is included. The RCA algorithms identify all cases with a diagnosis from pathology, thus, including all cancers is no more resource intensive than only selecting certain cancer types. The disadvantages of inviting all cancer types include enrolling small numbers of uncommon cancers that may not have sufficient sample size to study with sufficient power in this cohort alone. However, these cases can still be valuable for participation in disease-specific consortia studies [15, 16]. These cases could also be used to study multiple cancers defined by a specific exposure or similar treatment (e.g., studies of human papillomavirus-related cancers [17, 18], or studies of adverse outcomes from a specific chemotherapy regimen prescribed for multiple cancers [19]).

Our approach of using RCA algorithms facilitates the identification cancers that are rapidly fatal, and thus difficult to study. For example, our cohort currently includes 2917 lung cancer cases, 1371 pancreatic cancer cases, and nearly 8000 cases diagnosed at the distant summary stage (across all cancer types). Rapid identification and enrollment also provide an opportunity to collect blood specimens prior to the start of treatment; to date, we have 12,272 serum samples from cancer cases diagnosed after enrollment or within 6 months prior to enrollment. Our RCA approach has the disadvantage of missing up to 20% of cases. Most of these cases are missed because they are diagnosed through imaging or blood tests, instead of through histopathological evaluation, and thus do not have a pathology record at the time of diagnosis. The cases that are missed by the RCA algorithm are ultimately identified via the adjudication process with the tumor registries on average 6 months after diagnosis (but can lag up to 18 months) and invited to enroll in the cohort at that time. This lag in the tumor registry also contributes to the observed variability of the PPV.

While the cohort has many strengths, it is important to acknowledge the limitations, key among them are generalizability and the likelihood of survival bias. Our RCA methodology was designed to invite the participation of all KP members with a cancer newly diagnosed by biopsy. However, our response rate from the RCA process was only 9.8% overall, and notably lower among members of racial/ethnic groups other than non-Hispanic White, where the response rate was 14.6%. The results of our outreach to members with a history of cancer were similar; we had a 15.3% response rate among non-Hispanic Whites, but other racial/ethnic groups were below 5% (except for our “mixed/other” race category (5.2%)). Unfortunately, we did not explore the reasons why people declined to participate. It is likely that people diagnosed at an advanced stage, or otherwise with a poor prognosis, were less likely to enroll. Because members of racial/ethnic minority groups are often diagnosed at a later stage compared to Whites [20, 21], we may disproportionately under-represent advanced cancer cases among these groups. Importantly, we have over 20,000 incident cases who were diagnosed after enrollment in KPRB and this number will increase over time. While it is likely that the Cancer Cohort is not representative of the larger KP population, or cancer survivors across the U.S., we are able to query the EHR data available on all cases to understand the differences between people who enrolled in the cohort and those who did not, allowing for sample weights or other methods to statistically adjust for important biases that are identified.

### Future directions

Enrollment into the KPRB Cancer Cohort will continue at least through 2022, and the entire KPRB collection will be genotyped beginning in late 2021. Even while cohort recruitment is active, there are many opportunities to use this rich resource to address key questions in cancer control. A recent portfolio review of grants across the NIH identified important gaps in survivorship research [22]. Most research conducted to date has been among breast cancer survivors, thus, there is a need to understand the unique aspects of survivorship for other cancer types, and especially among men. There is a need to expand research on older and long-term survivors and assess the effectiveness of newer cancer therapies. Further, the Blue-Ribbon Panel established as part of the Cancer Moonshot developed a set of recommendations designed to exploit new advances in cancer prevention, diagnosis, and treatment [7]. Several recommended topics can be addressed within the KPRB Cancer Cohort, including retrospective analysis of biospecimens from patients treated with standard of care and a focus on cancer screening and risk reduction among cancer survivors, about 15% of whom will develop a new incident cancer [23, 24]. The report also noted the importance of including environmental, behavioral, and health-care resource factors in “big data” approaches modeling cancer etiology and outcomes.

## Conclusions

The application of precision medicine to cancer treatment must include not only genetic and molecular characterization, but consideration of lifestyle and health conditions that impact treatment efficacy, quality of life, and long-term survival. The KPRB Cancer Cohort is a rich data resource that will enable research to improve understanding of treatment efficacy and lifestyle factors that contribute to long-term cancer survival. Further, the cohort is diverse with respect to age, race/ethnicity and geographic location which will facilitate research on factors that contribute to the well-documented disparities in cancer survival [25, 26].

## Data Availability

Access to data used in this study may be obtained by application to the KPRB at kp.org/researchbank/researchers.

## Abbreviations

KPRB: The Kaiser Permanente Research Bank
EHR: Electronic health records
KP: Kaiser Permanente
RPGEH: Research Program on Genes, Environment and Health
IRB: Institutional Review Board
RCA: Rapid case ascertainment
SEER: Surveillance, Epidemiology and End Results
SNOMED: Systematized Nomenclature of Medicine
CAS: Cancer Alert system
ICD: International Classification of Diseases
NLP: Natural language processing
DCC: Data Coordinating Center
PPV: Positive predictive value
